# Targeted enrichment of the black cottonwood (*Populus trichocarpa*) gene space using sequence capture

**DOI:** 10.1186/1471-2164-13-703

**Published:** 2012-12-14

**Authors:** Lecong Zhou, Jason A Holliday

**Affiliations:** 1 Department of Forest Resources and Environmental Conservation, Virginia Polytechnic Institute and State University, 304 Cheatham Hall, Blacksburg, VA, 24061, USA

**Keywords:** Poplar, Sureselect, Exome, Population genomics

## Abstract

**Background:**

High-throughput re-sequencing is rapidly becoming the method of choice for studies of neutral and adaptive processes in natural populations across taxa. As re-sequencing the genome of large numbers of samples is still cost-prohibitive in many cases, methods for genome complexity reduction have been developed in attempts to capture most ecologically-relevant genetic variation. One of these approaches is sequence capture, in which oligonucleotide baits specific to genomic regions of interest are synthesized and used to retrieve and sequence those regions.

**Results:**

We used sequence capture to re-sequence most predicted exons, their upstream regulatory regions, as well as numerous random genomic intervals in a panel of 48 genotypes of the angiosperm tree *Populus trichocarpa* (black cottonwood, or ‘poplar’). A total of 20.76Mb (5%) of the poplar genome was targeted, corresponding to 173,040 baits. With 12 indexed samples run in each of four lanes on an Illumina HiSeq instrument (2x100 paired-end), 86.8% of the bait regions were on average sequenced at a depth ≥10X. Few off-target regions (>250bp away from any bait) were present in the data, but on average ~80bp on either side of the baits were captured and sequenced to an acceptable depth (≥10X) to call heterozygous SNPs. Nucleotide diversity estimates within and adjacent to protein-coding genes were similar to those previously reported in *Populus* spp., while intergenic regions had higher values consistent with a relaxation of selection.

**Conclusions:**

Our results illustrate the efficiency and utility of sequence capture for re-sequencing highly heterozygous tree genomes, and suggest design considerations to optimize the use of baits in future studies.

## Background

Population and quantitative geneticists interested in a wide variety of evolutionary questions are increasingly bypassing conventional genotyping strategies in favor of high-throughput re-sequencing. Tradeoffs between sample size and marker density can largely be overcome by these methods, making it possible to acquire dense genomic coverage in relatively large populations at a reasonable cost. A significant advantage of this approach is that it bypasses the conventional SNP discovery phase, ameliorating the problem of ascertainment bias and allowing for the scoring of SNPs without regard to *a priori* selection based on minor allele frequency. Several options exist for data acquisition in what can be generally called ‘genotyping-by-sequencing’ (GBS). While theoretically desirable, whole-genome re-sequencing may be too costly for species with large, complex genomes such as many plants, and may result in an unwieldy dataset in terms of the computational effort required for assembly and annotation. Genome complexity reduction provides an alternative, with the goal of reducing the genomic space that is sequenced [1-3]. Transcriptome sequencing (RNA-seq) is one option that has been available since the beginning of next-generation sequencing, and takes advantage of the natural ‘genome complexity reduction’ of the cellular transcriptional machinery [4,5]. RNA-seq has some advantages, most notably the ability to re-sequence much of the gene space, which is presumed to harbor a substantial fraction of the functional variation present in the genome. However, upstream regulatory regions are expected to harbor functional variants due to the potential for relatively minor changes to have profound effects on gene expression affecting fitness [3,6,7], and copy number polymorphisms with functional consequences cannot be scored from transcriptome data [8]. On the other hand, studies of neutral evolutionary processes (e.g., demographic history, population structure, gene flow) must avoid (or try to avoid) areas of the genome that may have been the targets of natural selection, and the ability to also score polymorphisms well outside of genes is therefore advantageous in such studies.

With these constraints in mind, two primary methods for genome complexity reduction have come to the fore in recent years: restriction enzyme-based approaches that recover a subset of the genome in a random but (mostly) repeatable manner, and sequence capture, in which specific genomic intervals are retrieved through hybridization of fragmented genomic DNA with labeled baits. The former class of methods have many variations, but all involve the use of one or more restriction enzymes to digest genomic DNA followed by size selection and sequencing [1,9-11]. The number of fragments isolated in this way depends on the choice of enzyme(s), and it is possible to enrich for regions that include transcribed genes by using an enzyme sensitive to methylation status. Due to the large size and repetitive content of many plant genomes, filtering out methylated heterochromatin prior to sequencing has been a popular way of enriching genomic libraries for gene-rich regions. This approach was initially applied Sanger-based EST sequencing [12], and subsequently to next-generation libraries [11]. Contemporary restriction-enzyme-based GBS is relatively inexpensive, avoids repetitive regions, and is amenable to situations where a limited number of haplotypes or extended linkage disequilibrium (LD) are expected, for example, in domesticated species or advanced pedigrees. That is, the goal of restriction-enzyme approaches is not necessarily to capture functional variants, but rather to saturate the ’haplotype space’ of the genome. This is similar in principal to conventional QTL mapping, but allows denser sampling of the genome and hence finer mapping of QTL in populations with lower LD than conventional backcross QTL-mapping pedigrees, but more LD than exists in wild populations.

Sequence capture involves designing long oligonucleotide ‘baits’ specific to regions of interest in the genome. In the original formulation, these baits were immobilized on a solid support, but solution-based capture using biotinylated-baits is more common at present [13-15]. The latter is more cost-effective and scalable for custom projects. While significantly more expensive than restriction-enzyme-based methods, sequence capture has a number of advantages for studies of genetic variation relevant to adaptation in large, unstructured, natural populations. These include more consistent and complete recovery and sequencing of the gene/exon space than restriction enzyme-based approaches. At the same time, sequence capture allows recovery of selectively neutral intergenic regions that can be used to estimate the effects of demographic processes such as migration history and population structure, a step that is usually required in association and landscape genomics studies. Another significant advantage of sequence capture is the ability to target long stretches of DNA such as entire exons or genes. Re-sequencing of longer fragments allows estimation of the site frequency spectrum and identification of cases of genetic hitchhiking – a hallmark of natural selection [16]. In general, for association mapping in trees, the haplotype-tagging approach relied on in human genetics may not be successful due to low LD in natural populations, and identifying functional variants requires denser genotyping [17-19]. However, while most long-lived tree species have very low LD, recent data from black cottonwood (*Populus trichocarpa*) suggest somewhat slower LD decay, on the order of a few kilobases, which may facilitate haplotype-tagging [20]. In balsam poplar (*P. balsamifera*), a close relative to *P. trichocarapa*, little average decay in LD was observed within genes [21].

**Figure 1:**
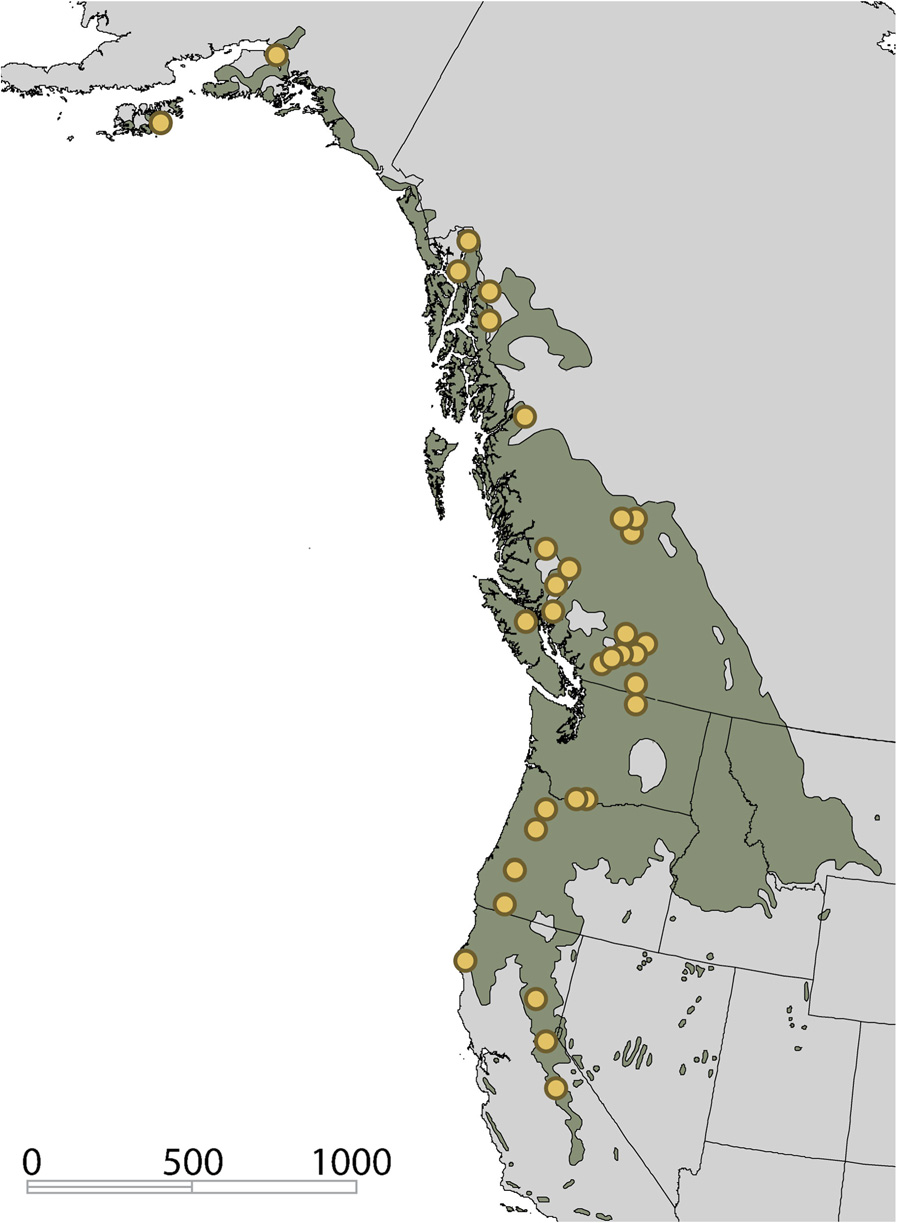
**Map of sampling locations.** Range of *P. trichocarpa* is indicated by green shading, and closed circles indicate origins of individual clones included in this study. Scale provided in units of kilometers.

Sequence capture was first used for targeted re-sequencing of the human genome [22-25], and is of particular current interest to studies of human disease, as it enables cost-effective genotyping of SNPs in large populations without *a priori* selection of SNPs based on minor allele frequency. While few reports exist at present, there is also considerable interest in the sequence capture approach for studies of complex traits in other taxa [26-29]. With large and complex genomes that harbor extensive repetitive elements, this technology is particularly useful in plants. Fu *et al*. [30] reported the application of a two-stage solid-state approach to the large and complex maize (*Zea Mays*) genome, in which libraries were first de-enriched of repetitive sequence, and then enriched for a 2.2Mb target region. Others have used both solution-based [31] and array-based [32] capture to successfully re-sequence target genes in big sagebrush (*Artemisia tridentata*) and soybean (*Glycine max*), respectively. Here we report the application of solution-based sequence capture to black cottonwood (also known simply as ‘poplar’). We targeted most of the poplar exome, as well as regions immediately upstream of genes (‘promoters’) and random genomic control intervals, and sequenced these in a preliminary panel of 48 individuals from across the natural range of the species (Figure 1). The success of this approach as well as design considerations for future sequence capture studies are discussed.

## Results

### Data preprocessing and alignment

We designed baits and completed hybrid capture of 20.76Mb of the *P. trichocarpa* exome, which included at least one exon from each predicted gene, as well as 320 randomly selected intergenic control regions. The 48 samples were pooled post-capture and sequenced in four lanes in a 2x100 paired-end format on an Illumina HiSeq instrument (12 samples per lane). For each clone we obtained an average of 12.48 million, 100bp reads, with a range of 9.49 to 14.78 million. An average of 86.7% (with a range of 83.9-90.0%) of the total raw reads passed our quality filters (see Methods section), and an average of 79.6% (with a range of 77.4-82.1%) of the raw reads remained in pairs after the preprocessing procedure (Additional file 1: Table S1). Read pairs for each clone were aligned to the unmasked poplar genome using the BWA alignment tool, and 97.1 ~ 98.8% of the quality-filtered reads were mapped onto the poplar genome. The vast majority of the reads (92.7 ~ 94.7% of the total inputs) were uniquely mapped (Additional file 2: Table S2), and those that did not were excluded from further analysis. One particular clone, SV08, had a much lower percentage of uniquely mapped reads, gapless alignments, and perfect alignments, and we therefore excluded this clone from further analysis.

### Efficiency of on-target enrichment

**Figure 2:**
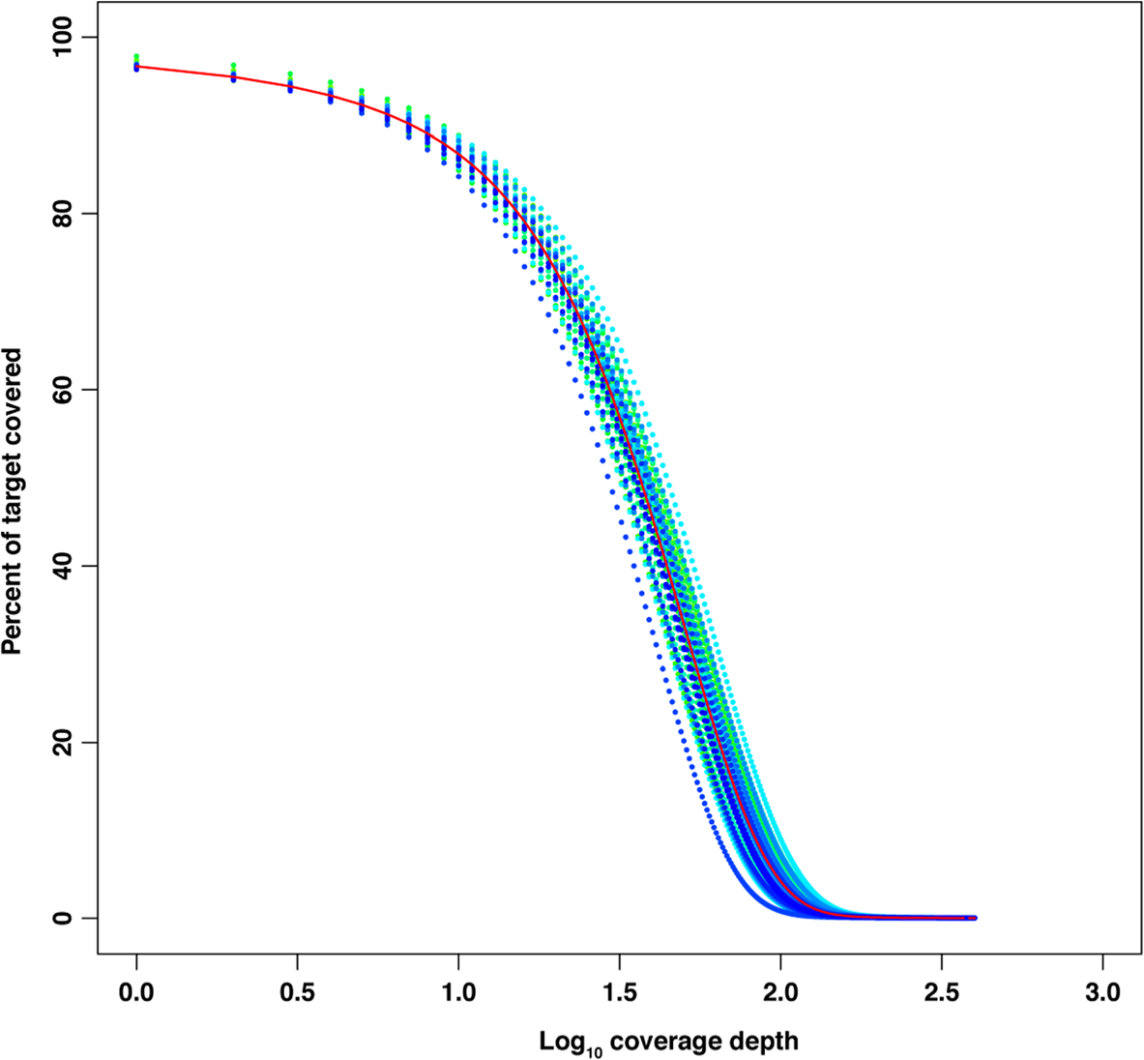
**Cumulative distribution of coverage depth in bait regions.** Both mean coverage across all 48 genotypes (red line) and mean coverage for individual genotypes (colored points) are provided.

Alignment of the reads to the poplar genome showed a high level of on-target enrichment efficiency (Figure 2). The 173k designed baits correspond to a total of 20.76Mb of genomic regions, which account for ~5% of the poplar genome. The number of baits per million base-pairs ranged from 47 to 858, with an average of 440. On average, 86.8% of base pairs in the bait regions were covered by uniquely mapped reads at ≥ 10X depth, and only 3.3% of the target regions were not covered. Due to the variable length of sheared DNA fragments in the prepped library, and because the median length of the sheared fragments (~150-200bp) was greater than the length of the baits, we acquired additional coverage in regions adjacent to baits. For analysis purposes, we defined these regions as up to 250 base pairs flanking both sides of the bait. On average, 37.7% of the adjacent area were covered at a depth ≥ 10X (Additional file 3: Figure S1). In contrast, little coverage was found in the off-target genomic regions, defined as those > 250 bp from any bait (Additional file 4: Figure S2). With a stringent cutoff of ≥ 10X coverage in all clones, we acquired data on 25.62Mb of the genome, whereas 64.09Mb of the genome was covered at ≥ 10X in at least one clone.

### Sequencing coverage in bait regions

**Figure 3:**
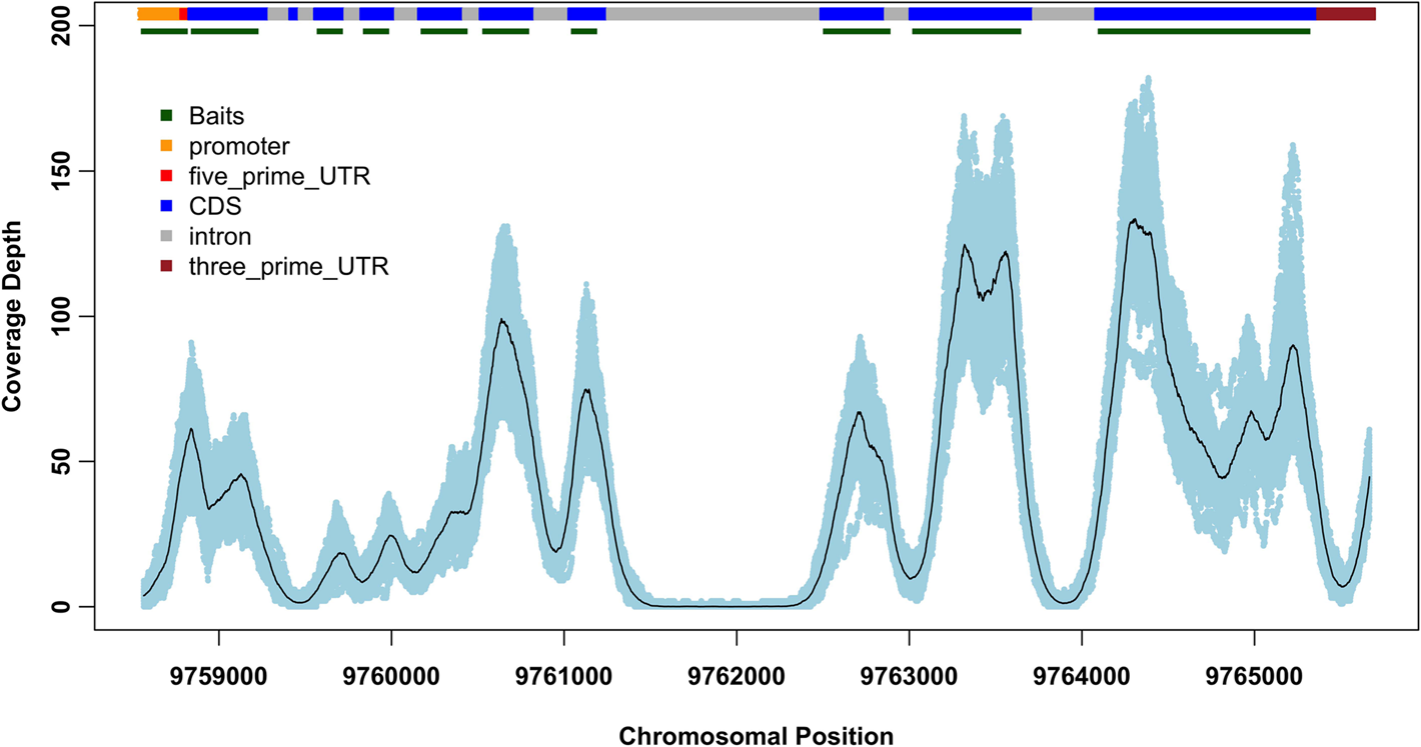
**Example of coverage depth for 47 clones across a single gene (POPTR_0006s12590) with multiple bait regions.** Gene features and bait positions are indicated. Exons less than 150bp were assigned a single bait, whereas multiple baits were placed end-to-end for longer exons.

Coverage among the 48 poplar clones was highly consistent and uniform in both target regions and their adjacent regions (Figure 2; Additional file 3: Figure S1). In target regions, the 47 clones (excluding SV08) had a narrow mean coverage range between 33X and 51X, with a mode ranging from 20-32X, and a median from 30-45X. Of the targeted bases, 67.4% were unanimously covered at least 10X, in addition, 18.2% of the base pairs within 250bp of either side of a given bait are also unanimously covered 10X or greater. To illustrate the depth of coverage within and adjacent to baits arrayed across the gene regions, we extracted coverage data for the gene model POPTR_0006s12590 as an example (Figure 3). Coverage depth for this gene, which was targeted by 30 baits, showed a consistent relationship between coverage depth and the positions of baits, and coverage was relatively uniform among clones.

### Coverage decay in regions adjacent to baits

**Figure 4:**
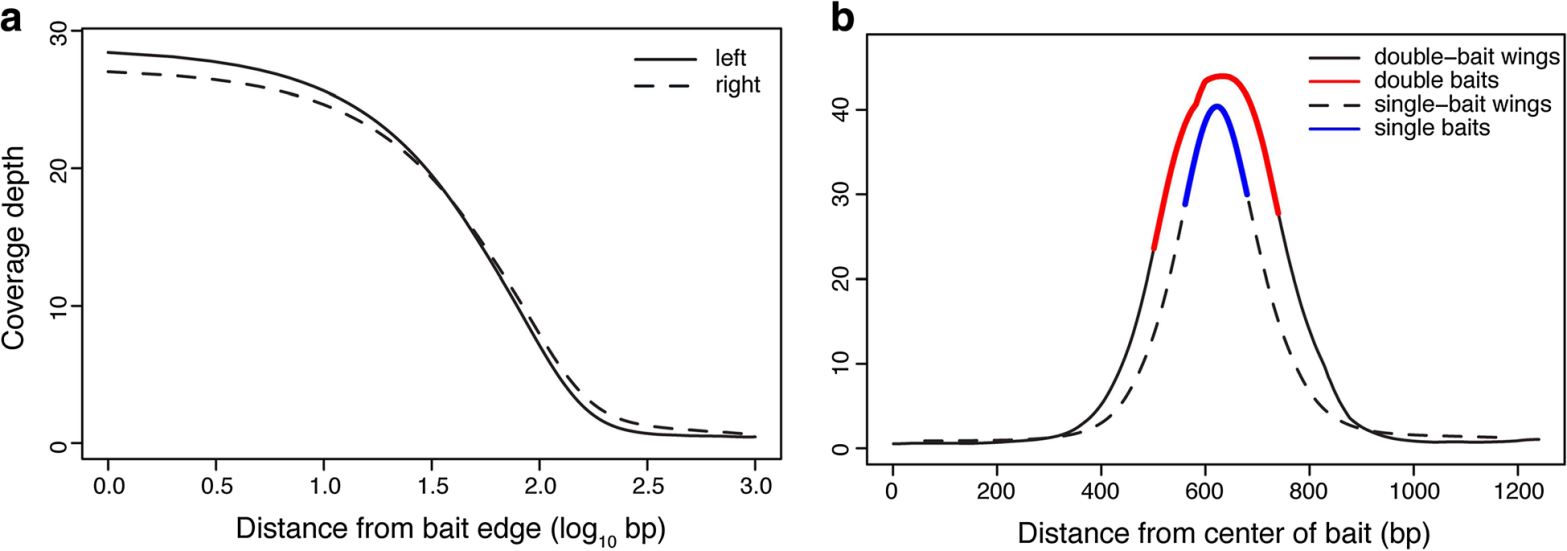
**Mean coverage depth as a function of distance from the 5**^**′ **^**(left) and 3**^**′ **^**(right) edges of bait regions (a) and coverage depth for target regions with single and double baits (b)**.

To investigate the decay in coverage moving away from the baits, we retrieved genomic regions adjacent to baits for which there was no neighboring bait within 1000bp. Sequencing depth in these ‘bait wings’ showed a consistent decay with distance from the bait (Figure 4a), with similar decay patterns between the left and the right wings. On average, approximately 80 base pairs nearest the bait were sequenced at a depth of 10X or more. We also extracted genomic regions that were covered by pairs of baits immediately adjacent to one another, and for which there was not another bait within 1000bp on either side. Interestingly, coverage analysis of these double-baits (Figure 4b) indicated a diminished effect on coverage in adjacent regions. On average, double-baits had a total of 386 bp (i.e., 240bp in the double baits and 146bp in the wings) that were covered at ≥10X, while single baits had 292bp (120bp on the bait and 172bp in the wings).

### Parameters affecting capture efficiency

**Figure 5:**
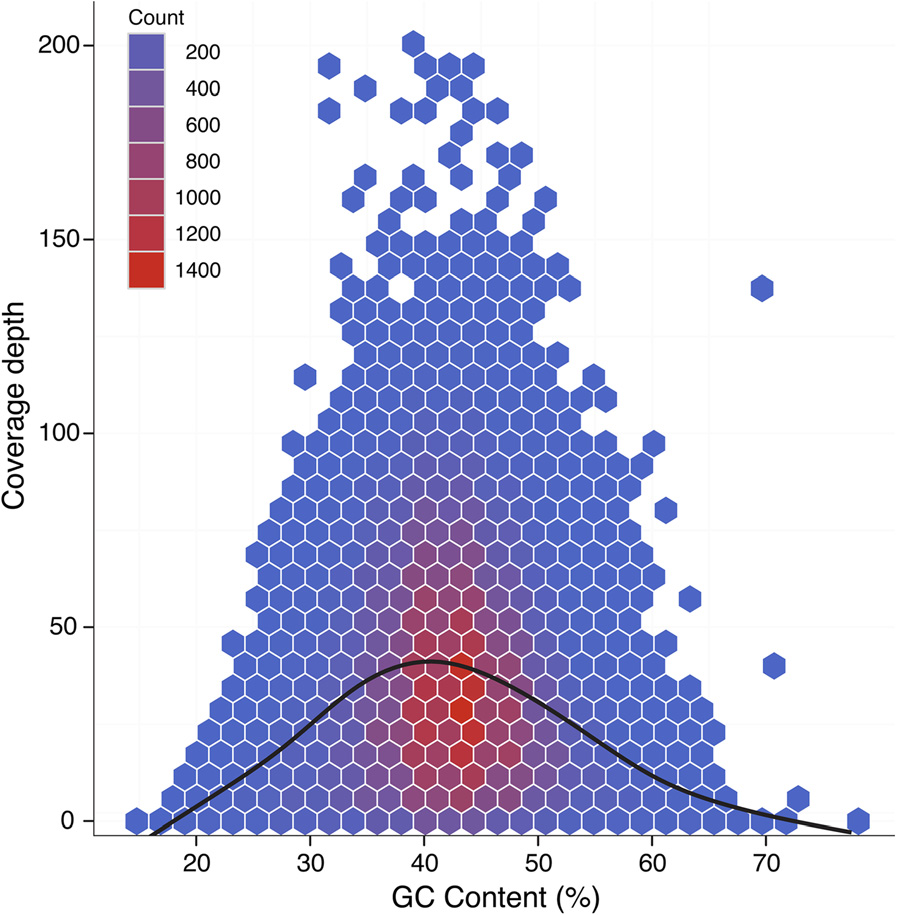
**Hexbin scatterplot and *loess *fit (black line) of the relationship between mean GC content of a target region and coverage depth.** Colors reflect the density of data points.

Although coverage depth for a given region was fairly uniform among clones (Figure 3), there was wide variation in coverage among target regions. As the algorithm Agilent Technologies uses to design baits is proprietary, we assessed the role that two variables played in capture efficiency: GC content and gene duplication. A clear relationship between GC content and coverage was apparent, with decreased coverage for target regions with both low and high GC content (Figure 5). To assess this relationship, we linearized the data by taking the absolute values of mean-subtracted GC content and found a highly significant but weak relationship with coverage depth (P < 0.001, R^2^ = 0.047). Another parameter that may also affect the recovery of target regions is the level of gene duplication. To determine if mean coverage depth and variance was affected by the presence of paralogs, we separated the data for target genes into two categories: single-copy and those with retained duplicates from the salicoid whole-genome duplication. Coverage depth was slightly higher for the duplicate class, while variance in depth of coverage was similar between these two categories (Additional file 5: Figure S3).

### SNP calling and diversity statistics

Our pipeline called a total of 1,129,874 SNPs (the union of 47 clones) that passed through the initial filter (≥10X coverage and SNP quality ≥ 30) for a particular clone. After applying additional filters (≥10X coverage in all the individuals and no insertion-deletion polymorphisms at the SNP loci in any individual), this SNP set was refined down to a total of approximately 495k candidate SNPs (Table 1). Of these, 240k candidate SNPs were from the targeted bait regions and 225k were from adjacent regions (i.e., within 500bp of a given bait), which combined accounted for 94.0% of the total. In terms of distribution of candidate SNPs in genomic features, 240k were from coding regions, 64k from promoter regions, and 1150 from the intergenic regions, with the rest distributed in introns and untranslated regions (Table 1).

**Table 1:** Number of called SNPs grouped by both genomic location class and positive relative to baits

	**Category**	**Candidate SNPS**	**Called SNPs**	**Percent of total**
Categorized by annotation of genomic region				
	Promoter	152223	64534	12.8
	5^′^-UTR	30586	13433	2.7
	Exon	446450	240693	47.9
	3^′^-UTR	29481	11753	2.3
	Intron	329559	149919	29.8
	Intergenic^1^	154785	22329	4.4
Categorized by position relative to baits				
	Within-bait	360558	241351	48.7
	Adjacent to baits	568115	225581	45.5
	Off-target	201201	28594	5.8

^1^Inclues SNPs from both targeted intergenic control regions as well as off-target regions.

We used MANVa software (http://www.ub.edu/softevol/manva/) to calculate multi-locus nucleotide diversity and Tajima’s D, a summary statistics of the folded site frequency spectrum, for the 320 intergenic regions, as well as 40,668 gene regions. For both of these, flanking regions were incorporated where coverage was sufficient. Gene regions included upstream and downstream flanking sequence, as well as introns and UTRs. We merged gene regions in cases where two or more genes were adjacent in the genome and depth of coverage between them was sufficient. Per site nucleotide diversity, estimated using Watterson’s θ and Tajima’s π, was higher in intergenic regions – 0.0071 and 0.0064, respectively, compared with 0.0035 and 0.0029, respectively, for the genic regions (Table 2). The difference between these two estimators of nucleotide diversity yielded a mean negative Tajima’s D, which was more negative in genic regions (D = −0.327) compared with intergenic regions (D = −0.181). As genes with retained duplicates from the salicoid whole-genome duplication may be difficult to accurately align, which could lead to higher levels of false-positive SNPs, we assessed diversity in single-copy genes vs those with retained duplicates. Diversity was similar between these two groups, though slightly higher for single-copy genes (Table 2).

**Table 2:** Average multilocus diversity estimates for genic (including introns and adjacent captured sequence) and intergenic regions. Associated standard deviations are given in parentheses

**Regions**	**Length**	**S**	**θ**	**π**	**D**
All genes	344 (239)	6.2 (5.8)	0.0035 (0.0028)	0.0029 (0.0031)	-0.327 (0.872)
Single copy genes	330 (251)	6.0 (5.3)	0.0036 (0.0027)	0.0030 (0.0032)	-0.308 (0.940)
Duplicated genes	363 (219)	6.0 (5.4)	0.0032 (0.0023)	0.0027 (0.0027)	-0.339 (0.931)
Intergenic	271 (114)	9.9 (6.4)	0.0071 (0.0052)	0.0064 (0.0046)	-0.181 (0.864)

Abbreviations: S, segregating sites; θ, Watterson’s theta; π, Tajima’s pi; D, Tajima’s D.

## Discussion

While contemporary high-throughput sequencing technologies make genome-wide studies feasible in both model and non-model species, whole-genome re-sequencing is usually still cost prohibitive. Even where funds are available, studies of natural variation and its relationship to adaptation may be better served by increasing sample sizes, rather than genomic coverage beyond a certain point. Our data suggest that sequence capture results in a dataset that is a reasonable tradeoff between cost and genotyping coverage in regions most likely to contribute to adaptation – that is, the gene and regulatory space.

### Capture efficiency and specificity

Our results suggest that sequence capture is a reliable approach to score SNPs in both the gene space and non-repetitive intergenic regions. Although the depth of coverage varied among target regions, it was consistent among clones for a given bait. Only about 3% of the sequence data did not map to a target region, suggesting that baits did not frequently hybridize with off-target fragments. The only exception to this was for a single clone collected from north central California, USA (SV08), which had a much lower percentage of mapped reads. Preliminary population structure analysis suggests that this clone clusters separately from all of our other samples, and is likely either another species – *P. fremontii* or a *P. trichocarpa* x *fremontii* hybrid (data not shown).

It should be noted that the level of off-target capture in our study may be low in part because we designed baits for most of the poplar gene space. Hence, off-target hybridization of one of our baits would require a level of complementarity with the off-target region that is probably rare given the 120bp length of the baits. By contrast, more focused studies may observe inflated off-target capture in cases where the goal is to retrieve, for example, specific members of gene families. In such cases, recovery of untargeted paralogous genes may be expected. This is a particular issue in *Populus*, which has experienced a relatively recent whole genome duplication [33]. As sequence capture baits have some tolerance for mismatches, it is likely that a bait designed for a given gene will capture paralagous genes in cases where sequence divergence is not high. While this is not a problem in the context of whole exome capture, it may present a problem for accurate assembly of the resulting short-read sequence data. To evaluate this, we separated genes into two categories – those with retained paralogs from the salicoid whole-genome duplication event, and those without paralogs (based on the list provided in Rodgers-Melnick et al. [34]). If reads from a given gene were assembling to their cognate paralog, and vice-versa, we would expect a higher rate of putative SNPs in the ‘duplicate’ category of genes. This was not the case in our data – both the single-copy and duplicate categories had similar rates of SNP discovery – which suggests that our assembly pipeline handled reads from duplicate genes reasonably well.

### Nucleotide diversity

Our SNP discovery pipeline called approximately 495,000 SNPs, although many more possible polymorphisms were detected before we applied rigorous thresholds for accepting a polymorphism. As we recovered 25.62 Mb of sequence data including both target and adjacent regions, this corresponds to about one SNP per 52bp. This value is higher than that found in recent black cottonwood and balsam poplar studies [21,35,36], but similar to trembling aspen (*P. tremuloides*) [37] and European aspen (*P. tremula*) [38]. Slavov et al. [35] recently reported genome-wide resequencing of the poplar genome for 16 individuals originating from diverse locations across the range. For this panel, one SNP per 313bp were detected (using similar quality-score (QS) filtering methods to those that we employed). Geraldes et al. [36] reported a higher SNP rate – approximately one per 142bp – based on transcriptome re-sequencing of a panel of *P. trichocarpa* individuals that was similar to that of Slavov et al. in terms of geographic diversity (though with the notable addition of several clones from near the northern range limit in the northwest tip British Columbia). That the SNP rate obtained by Geraldes et al. is substantially lower than our own may reflect that fact that they studied only expressed sequences, which are more likely to be constrained by natural selection. Probably more important, our population was about 2x larger and included individuals from relatively isolated populations at the northern range limit on the south coast of Alaska.

As SNP discovery rates per base-pair depend on the size and diversity of the re-sequenced panel of individuals, we calculated per site nucleotide diversity – estimated by Watterson’s θ and Tajima’s π – to allow us to compare the variation in our data more directly with studies of other *Populus* species. Tajima’s π for gene regions was very similar to values obtained from a large Sanger-based re-sequencing study of *P. balsamifera*[21], a close relative of *P. trichocarpa*, and ~30% lower than for European aspen (*P. tremula*) [38]. Intergenic regions carried about two-fold higher diversity than the gene regions, and data from the poplar genome sequence [33] support this result – the number of SNPs in the intergenic space in the sequenced Nisqually-1 clone was about double that of exons. That nucleotide diversity in our data was higher for non-coding regions far from genes suggests a relaxation of purifying/background selection compared with gene regions. The difference between our estimates of θ and π resulted in a slightly negative average Tajima’s D, which was more pronounced in genes compared with intergenic regions, suggesting that selection is relaxed in the intergenic space. Deviations from the neutral expectation for summary statistics of the site frequency spectrum (e.g., Tajima’s D) are common among forest trees [38-41]. Simulations have shown that such results are consistent with changes in population size during Pleistocene range expansion and contraction, and our data suggest that similar demographic processes may have affected the genome-wide frequency distribution of mutations in *P. trichocarpa*[38,39,42,43]. Future studies of the genetic targets of natural selection in this species will therefore need to account for this genome-wide deviation from the neutral expectation for the site frequency spectrum.

### Capture and alignment for duplicated genes

Paralagous genes in the poplar genome that arose from the salicoid whole-genome duplication event, as well as tandem gene duplication, may create problems both for the physical process of capturing fragments of genomic DNA, as well as correctly aligning the resulting sequence data. Because the solution hybridization process tolerates some mismatch, baits designed for one gene may capture close paralogs. This is not a problem for whole-exome sequencing so long as the resulting data can be aligned correctly to the genome. However, in cases where baits are designed against two paralagous genes, it is possible that hybridization and capture could be skewed to one or the other gene. Such a skew should increase mean coverage depth in duplicated vs. single-copy genes – though fewer genes will be captured, those that are will be captured in greater abundance and hence sequenced to a greater depth. At the same time, capture bias should result in a greater variance in the duplicated vs. single-copy genes – because capture bias is unlikely to be complete, some genes will be ‘over-captured’ and hence sequenced to great depth, whereas others will be ‘under-captured’ and sequenced only lightly. We did not observe either of these effects – both mean depth of coverage and variance were similar between single-copy and duplicate gene pairs – and we therefore conclude that capture efficiency in our study was not substantially impacted by the presence of paralagous genes. With respect to the second issue noted above – the impact of paralogs on the ability to correctly align sequence data – we used estimates of nucleotide variability in these two classes of genes to determine if there was a significant problem with misalignment of genes to their respective paralogs in the genome. Such misalignment should increase the level of SNPs in the duplicate category, as fixed differences between duplicates would be incorrectly called as SNPs. Our estimates of nucleotide diversity for single-copy vs duplicates were very similar, which suggests that misalignment was not a problem. Interestingly, diversity was slightly higher in the single-copy class of genes (π = 0.0030 vs. 0.0027 for genes with duplicates), and Tajima’s D was somewhat less negative (−0.308 for single-copy vs. -0.339 for duplicates). This result suggests that genes with duplicates are slightly more constrained by selection than single-copy genes, which is counterintuitive. While this may simply be a stochastic effect, more detailed analyses of these data are warranted to elucidate what, if any, evolutionary mechanism may be at work here.

### Possible improvement to future bait design

Particularly for whole exome sequencing in non-model organisms – i.e., custom bait designs – the cost of the bait pools may limit the size of targeted regions to something less than the cumulative length of the exome, as was the case in the current study. One possible way to circumvent this issue is to ligate index adapters and pool samples before capture, thereby allowing for the capture of a larger number of samples than a given kit was designed for. Kirst et al. [44] reported success with this approach, although a greater proportion of off-target capture may be expected. Pre-capture pooling is now supported by both Agilent Technologies and Roche NimbleGen (SeqCap EZ platform; http://www.nimblegen.com/seqcapez/), and this approach both reduces liquid handling as well as reagent costs.

**Figure 6:**
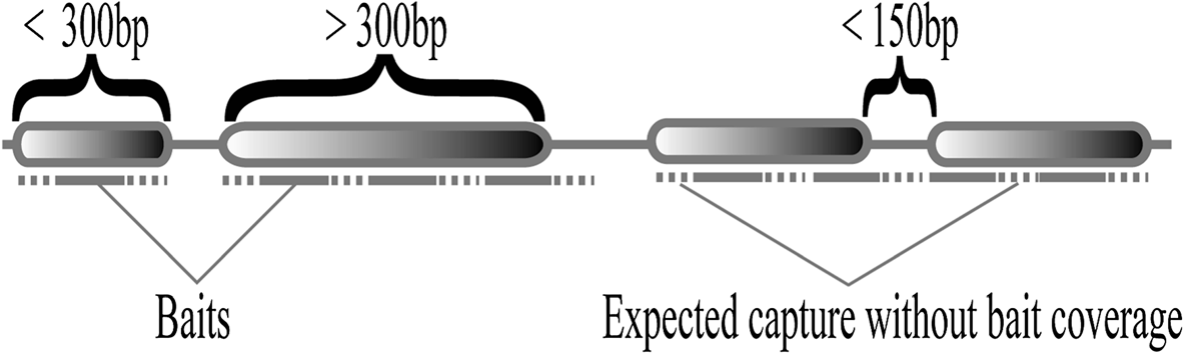
**Proposed strategy to optimize bait usage for maximal coverage of target regions while minimizing the size of the bait pool.** Solid lines indicate bait placement, and dotted lines indicate regions for which acceptable depth of coverage is expected in the absence of bait coverage. For target regions less than 300bp, a single bait is placed centered on the region. For targets greater than 300bp, baits are tiled with ~150bp gaps between adjacent baits. When two target regions are separated by less than 150bp, a contiguous sequence is expected to be recovered in the absence of bait coverage between the two regions.

Our results suggest improvements that can be made in bait design to recover the largest fraction of the gene space given a particular capture size (kits vary from several hundred Kb to tens of Mb). Specifically, we found that on average approximately 80bp on either side of a given 120bp target region returned sequence of acceptable depth to call SNPs. This is not a surprising result given that we sheared our genomic DNA preparations to an average length of ~200bp. As a result, hybridized genomic DNA fragments were longer than the baits that retrieved them. Future studies that employ sequence capture would therefore benefit from the following changes to bait design when the goal is to maximize the length of captured regions while minimizing the expense of the bait pools: (i) for target regions less than ~300bp, a single bait should be centered on the target – most or all of the flanking regions will be recovered; (ii) for target regions >300bp, gaps of ~150bp should be arrayed between the targeted regions; and (iii) when two target regions are separated by <150bp, they can be treated as one, with gaps as in (ii) allowed to fall at whatever location the target sequence dictates (Figure 6).

With respect to depth of coverage, we acquired an average of ~40X with 12 indexed samples in each lane, sequenced in a 2x100bp format on an Illumina HiSeq instrument. It may be possible to multiplex more samples within a single lane depending on the goals of the study and the analytical methods used. With the relatively high LD recently reported in *Populus*[20], imputation of missing data may be a reasonable strategy. However, as our results show, coverage depth from captured libraries is both heterogeneous among regions and uniform across samples for a particular region. While we show that GC content plays a role in coverage depth for a given target, other unknown factors are more important. It is therefore difficult to assess *a priori* which targets will exhibit poor capture and hence lower coverage depth. With lighter coverage, a greater proportion of regions will have low or no coverage. This missing data will likely be missing in all samples for a given target region, thus making it impossible to impute missing data for such targets. Depending on the purpose (e.g., association mapping) and level of LD in the focal species, this may be acceptable. It should be noted that the strategy for bait design must be considered when calculating the approximate mean coverage depth expected from a given multiplex sequencing scenario. The strategy outlined above is designed to maximize the cumulative length of captured regions, and the amount of captured sequence may be double the size of the bait design.

## Conclusions

The goal of this study was to test solution sequence capture as a means to genotype the exome of the highly heterozygous model tree *P. trichocarpa*. We targeted nearly 21Mb of the genome, and with 12 samples in a single lane of an Illumina HiSeq instrument, approximately 85% of our target regions returned quality data of acceptable depth to call SNPs. In addition to the target region, we recovered ~80bp of flanking sequence on either side of baits. Our results show that solution sequence capture is a reliable method to enrich the gene space in complex plant genomes. A relatively small proportion of off target capture was observed, and inter-sample variability in depth of coverage for a given locus was small. With careful attention to bait design, it should be possible to recover approximately double the amount of sequence targeted due to the recovery of flanking regions, though it is important to account for this additional sequence when determining multiplex level for sequencing.

## Methods

### Plant material and DNA extraction

Cuttings from 48 poplar clones were collected from geographic locations of diverse latitude, longitude and elevation (Figure 1). The cuttings were rooted in a 3:2 mixture of peat:perlite and kept in a greenhouse at ambient temperature of 20°C throughout the rooting process. The ramets were misted twice daily for 30min to limit transpirational water loss. For DNA extraction, young leaves were sampled from an individual plant and immediately flash frozen in liquid nitrogen before being stored in low-temperature freezer (−80°C). DNA was extracted from the leaves using Qiagen DNeasy® Plant Mini Kit following the standard protocol with minor modifications as follows: starting material was increased to a maximum of 500mg, buffers volumes were scaled-up proportionally to the increased tissue used, and the column was eluted twice each with 75 μl 1x low TE buffer. DNA quality was checked in a NanoDrop Spectrophotometer, and a 260/280 ratio of 1.7-2.0 with a minimum concentration of 20 ng/μl were applied as cutoffs for acceptable extractions.

### Probe design and capture library synthesis

Phytozome version 7.0 annotation and assembly files for black cottonwood [33] (corresponding to assembly version 2.2 of the poplar genome) were downloaded (http://www.phytozome.org/), and used in design of short genomic regions (commonly called baits) targeting exons, promoter, and intergenic control regions. The poplar genome assembly has a total of 2518 scaffolds, with the first 19 scaffolds corresponding to the poplar chromosomes. For simplicity and convenience, we generated an artificial assembly (“chromosome 20”) by sequentially concatenated all the small scaffolds (i.e., Scaffold 20 to 2518). A total of 173,040 baits of 120bp in length were designed using SureSelect eArray software (Agilent Technologies, Santa Clara, California, USA), which covered a total of 20.76Mb genomic regions (or ~5.0% of the entire genome; Additional file 6: Figure S4). The baits targeted more than 39,000 of the 40,668 loci containing protein-coding transcripts. Approximately 145,000 baits targeted the exons, 26,000 targeted 250bp regions upstream of the genes (i.e., putative promoter regions), and 600 baits targeted intergenic regions to be used as putative selectively neutral control regions for population genetic analyses. These control regions were roughly evenly distributed across chromosomes, and selected at random from non-repetitive intergenic areas of the genome. As the cumulative length of the predicted exons exceeded the available baits, following bait design in eArray we looped through the gene list, selecting one bait for each gene at each pass, until the maximum number of baits was reached. Priority was given to a small subset of 3100 genes (or 8% of the total) corresponding to Gene Ontology terms relevant to climatic adaptation, to which ~30,000 baits (or 18% of the total) were assigned. For these, baits corresponding to all exons were retained. Using this design, a custom biotinylated RNA bait library was synthesized by Agilent Technologies.

### Library preparation

Our targeted enrichment was based on Agilent’s SureSelect^XT^ target enrichment system for Illumina paired-end sequencing. The 48 samples were randomly assigned to 4 groups, each of which corresponding to a HiSeq sequencing lane, and the 12 samples in each group were randomly assigned to an index (1 to 12). The prepped library for each clone was prepared according to the Agilent SureSelect^XT^ protocol (version 1.2). Briefly, 3.0 μg of poplar genomic DNA (in 130 μl 1X Low TE Buffer in a 1.5-mL LoBind tube) was sheared on a Covaris S220 instrument at the University of Georgia Genomics Core, followed by ends repair, 3^′^-end adenylation, adaptor ligation, and amplification. Agencourt AMPure XP beads were used to purify the libraries following each step. Library quality was assessed using a BioRad Experion with DNA 1K chips.

### Target enrichment

680 ng (200 ng/ μl) of the prepped libraries above were used in the solution hybridization to the RNA baits, which was carried out at 65°C according to the Agilent protocol in a PCR machine (Eppendorf Mastercycler gradient). Following hybridization, target regions were purified on magnetic beads followed by post-hybridization amplification of the captured library to add index sequences.

Captured libraries were quantified using real-time PCR and a NanoDrop Spectrophotometer, and quality-checked on an Agilent Technologies Bioanalyser (DNA-7500 chip). Libraries were pooled such that each index-tagged sample was present in equimolar amounts, with final concentration of the pooled samples of 50nM. The pooled samples were subject to cluster generation and sequencing using an Illumina HiSeq 2000 System in a 2x100 paired-end format at the David H. Murdock Research Institute (Durham, North Carolina, USA).

### Data analysis and SNP detection

Analysis of the 100bp HiSeq sequencing reads involved custom pipelines to preprocess the raw data, align to the poplar reference genome, and identify putative single nucleotide polymorphisms (SNPs). The pipelines were Perl wrappers and necessary custom programming scripts in Perl, R and Shell, which piped public software tools for quality-control, assembly, and SNP discovery (see below). Preprocessing of raw data included quality control, verification of de-multiplexing, trimming the reads when necessary, and re-pairing. A high quality read met the following criteria: properly de-multiplexed (up to 1 mismatch was allowed in the barcode), mean quality score (Q-score) ≥ 30, minimum Q-score ≥ 13, and zero undetermined nucleotides. If an undetermined base was present near either end of a read, it was trimmed and passed to downstream analysis if the rest of the read met the above criteria. As a last step, the QC-passed reads were re-paired for alignment.

Only reads that passed the above QC thresholds were aligned to the reference genome of *P*. *trichocarpa*, which consisted of 19 chromosomes and the one concatenated scaffold described above. The Burrows-Wheeler Aligner (BWA) [45], a fast and accurate short read alignment tool that employs a Burrows-Wheeler transform (version 0.6.1), was used to align our short reads. The reference genome was indexed using the IS linear-time algorithm. For each poplar clone, the paired-end reads were aligned separately using the *aln* command with default settings, and then converted to alignments in the SAM format in pairs via the *sampe* command.

SNPs were called using SAMtools [46] by first converting uniquely aligned SAM-formatted records into a binary format, and then sorting and indexing the resulting files. For each clone, the *mpileup* function (with input options “-P ILLUMINA -C50 -ugf” enabled to specify the platform and the coefficient for downgrading mapping quality, and to compute genotype likelihoods) was subsequently applied to generate raw variants which were thereafter converted into variant call format (VCF) [47] with the *bcftools* function in SAMtools. Putative SNP variants were only considered if they had at least 10X coverage and a quality score ≥30, which indicates a 1 in 1000 chance of error. BEDTools [48] was used to calculate the genome and regional coverage for each of the clones. The coverage of the entire genome was calculated for each uniquely mapped alignment (one file per clone) using *genomeCoverageBed*. For coverage of regions of interest, *bed* files for the regions (eg., baits, regions adjacent to baits, promoter and gene regions) were generated and then, *coverageBed* was used to calculate the coverage depth. Finally, we further filtered out those variants called from inconsistently covered regions such that only SNPs from genomic regions that were unanimously covered at ≥ 10X in all the *P. trichocarpa* clones were selected as candidate SNPs. Using this SNP set, we calculated diversity parameters and summary statistics for the site frequency spectrum using MANVa software (http://www.ub.edu/softevol/manva/).

To investigate coverage decay with increasing distance from baits, we retrieved genomic regions adjacent to baits for which there was no neighboring bait within 1000bp (‘single baits’). Similarly, we also extracted genomic regions that were covered by pairs of baits immediately adjacent to one another, and for which there was not another bait within 1000bp on either side. Here, we refer to these bait-spanning regions as “wings”.

Finally, to assess a possible relationship between GC content and capture efficiency, we subset the single baits that are apart from any other baits by at least 120bp and then calculated mean read depth across all samples (excluding one clone with a low percentage of mapped reads – see Results section) as well as the percentage of guanine and cystosine residues in each target region. The results of this analysis were displayed using the *ggplot2* package in R (*geom_hex* and *stat_smooth* functions).

#### Data availability

The HiSeq data discussed in this publication have been deposited in NCBI’s Sequence Read Archive (SRA) (http://www.ncbi.nlm.nih.gov/Traces/sra/). The SRA accession number is SRA058855.

## Competing interests

The authors declare no competing interests.

## Authors’ contributions

JH designed the study, LZ collected and analyzed the data, and JH and LZ wrote the paper. Both authors read and approved the final manuscript.

## Supplementary Material

Additional file 1 **Table S1.** Statistics of raw and preprocessed reads. Click here for file

Additional file 2 **Table S2.** Statistics of sam alignments. Click here for file

Additional file 3 **Figure S1.** Cumulative distribution of coverage depth in adjacent regions adjacent to baits. Both mean coverage across all 48 genotypes (red line) and mean coverage for individual genotypes (colored points) are provided. Click here for file

Additional file 4 **Figure S2.** Cumulative distribution of coverage depth in off-target regions. Both mean coverage across all 48 genotypes (red line) and mean coverage for individual genotypes (colored points) are provided. Click here for file

Additional file 5 **Figure S3.** Boxplots of coverage depth for single-copy genes and those with retained salicoid duplicates. Click here for file

Additional file 6 **Figure S4.** Distribution of baits across the 19 poplar linkage groups. Points indicate bait locations, light blue line indicates number of baits in 1Mb sliding windows, and red line indicates mean depth of sequencing coverage in 1Mb sliding windows. Click here for file
