# Supplementary material for: Targeted enrichment of the black cottonwood (Populus trichocarpa) gene space using sequence capture
Source: BMC Genomics. 2012 Dec 14;13:703. doi: 10.1186/1471-2164-13-703 (PMC3542275; doi:10.1186/1471-2164-13-703)
Supplement: Additional file 1 — Table S1. Statistics of raw and preprocessed reads. [file 1471-2164-13-703-S1.docx]

| Supplemental Table 1. Statistics of raw and preprocessed reads | | | | | | | | | | | | |
| --- | --- | --- | --- | --- | --- | --- | --- | --- | --- | --- | --- | --- |
| **Lane** | **Read** | **Stat** | **No. good full length reads** | **No. scavenged reads** | **No. low quality reads** | **No. N-carrying reads** | **No. bar-code errors** | **Total reads** | **No.**  **good**  **reads** | **% good reads** | **No.**  **paired good reads** | **%**  **paired reads** |
| 1 | 1 | min | 8,881,583 | 604,266 | 1,321,931 | 2,884 | 0 | 10,831,644 | 9,485,849 | 87.6 | 8,585,130 | 79.3 |
|  |  | max | 11,743,074 | 772,565 | 1,699,921 | 4,038 | 0 | 14,219,465 | 12,515,639 | 88.2 | 11,344,093 | 80.0 |
|  |  | avg | 10,363,676 | 684,107 | 1,501,345 | 3,436 | 0 | 12,552,565 | 11,047,784 | 88.0 | 10,017,857 | 79.8 |
| 1 | 2 | min | 8,592,097 | 625,722 | 1,562,052 | 45,504 | 0 | 10,831,644 | 9,224,088 | 85.2 |  |  |
|  |  | max | 11,339,497 | 813,142 | 2,007,304 | 59,522 | 0 | 14,219,465 | 12,152,639 | 85.7 |  |  |
|  |  | avg | 10,013,491 | 717,865 | 1,768,578 | 52,631 | 0 | 12,552,565 | 10,731,356 | 85.5 |  |  |
| 2 | 1 | min | 7,740,680 | 2,103,734 | 1,479,862 | 3,598 | 0 | 11,327,874 | 9,844,414 | 86.6 | 8,918,330 | 78.3 |
|  |  | max | 10,026,218 | 2,715,534 | 1,877,075 | 4,589 | 0 | 14,623,416 | 12,741,752 | 87.2 | 11,517,038 | 78.9 |
|  |  | avg | 9,077,712 | 2,462,834 | 1,714,511 | 4,144 | 0 | 13,259,201 | 11,540,545 | 87.0 | 10,437,032 | 78.7 |
| 2 | 2 | min | 8,945,360 | 677,632 | 1,662,729 | 42,153 | 0 | 11,327,874 | 9,622,992 | 84.6 |  |  |
|  |  | max | 11,520,499 | 873,147 | 2,174,462 | 55,308 | 0 | 14,623,416 | 12,393,646 | 85.0 |  |  |
|  |  | avg | 10,457,469 | 788,454 | 1,963,066 | 50,212 | 0 | 13,259,201 | 11,245,923 | 84.8 |  |  |
| 3 | 1 | min | 8,884,002 | 685,930 | 1,439,894 | 7,156 | 0 | 11,054,066 | 9,588,857 | 86.4 | 8,622,338 | 77.4 |
|  |  | max | 11,898,399 | 924,342 | 1,943,433 | 9,582 | 0 | 14,775,756 | 12,822,741 | 86.9 | 11,557,464 | 78.2 |
|  |  | avg | 10,369,401 | 812,700 | 1,705,810 | 8,350 | 0 | 12,896,262 | 11,182,101 | 86.7 | 10,057,958 | 78.0 |
| 3 | 2 | min | 7,369,675 | 1,955,749 | 1,663,679 | 40,741 | 0 | 11,054,066 | 9,325,424 | 83.9 |  |  |
|  |  | max | 9,893,221 | 2,628,936 | 2,198,167 | 55,432 | 0 | 14,775,756 | 12,522,157 | 84.7 |  |  |
|  |  | avg | 8,607,563 | 2,288,208 | 1,952,600 | 47,892 | 0 | 12,896,262 | 10,895,770 | 84.5 |  |  |
| 4 | 1 | min | 8,077,924 | 465,139 | 942,981 | 3,810 | 0 | 9,489,854 | 8,543,063 | 89.5 | 7,780,722 | 81.7 |
|  |  | max | 10,298,004 | 599,212 | 1,216,812 | 5,008 | 0 | 12,113,677 | 10,896,106 | 90.0 | 9,940,187 | 82.1 |
|  |  | avg | 9,498,558 | 555,879 | 1,128,008 | 4,603 | 0 | 11,187,048 | 10,054,437 | 89.9 | 9,168,843 | 82.0 |
| 4 | 2 | min | 7,760,466 | 485,222 | 1,205,377 | 38,789 | 0 | 9,489,854 | 8,245,688 | 86.7 |  |  |
|  |  | max | 9,937,268 | 613,235 | 1,516,002 | 49,442 | 0 | 12,113,677 | 10,548,233 | 87.2 |  |  |
|  |  | avg | 9,157,647 | 571,044 | 1,412,717 | 45,640 | 0 | 11,187,048 | 9,728,691 | 87.0 |  |  |
| Avg | 1&2 | min | 7,369,675 | 465,139 | 942,981 | 2,884 | 0 | 9,489,854 | 8,245,688 | 83.9 | 7,780,722 | 77.4 |
|  |  | max | 11,898,399 | 2,715,534 | 2,198,167 | 59,522 | 0 | 14,775,756 | 12,822,741 | 90.0 | 11,557,464 | 82.1 |
|  |  | avg | 9,693,190 | 1,110,136 | 1,643,329 | 27,113 | 0 | 12,473,769 | 10,803,326 | 86.7 | 9,920,423 | 79.6 |
