# Supplementary material for: Targeted enrichment of the black cottonwood (Populus trichocarpa) gene space using sequence capture
Source: BMC Genomics. 2012 Dec 14;13:703. doi: 10.1186/1471-2164-13-703 (PMC3542275; doi:10.1186/1471-2164-13-703)
Supplement: Additional file 2 — Table S2. Statistics of sam alignments. [file 1471-2164-13-703-S2.docx]

| Supplemental Table 2. Statistics of sam alignments in 48 poplar clones | | | | | | | | | | | |
| --- | --- | --- | --- | --- | --- | --- | --- | --- | --- | --- | --- |
| **Clone** | **No.**  **reads** | **No. alignments** | **No. distinct sequence** | **No. mapped reads** | **% mapped reads** | **No. uniquely mapped reads** | **%**  **uniquelymapped_reads** | **No.**  **gapless alignments** | **%**  **gapless alignments** | **No. perfect alignment** | **%**  **perfect alignment** |
| BE02 | 11344093 | 22688186 | 18514637 | 11150851 | 98.3 | 10671703 | 94.1 | 21200241 | 93.4 | 15657609 | 69.0 |
| BE05 | 10609813 | 21219626 | 17535576 | 10410901 | 98.1 | 9960940 | 93.9 | 19774483 | 93.2 | 14614131 | 68.9 |
| TA00 | 8585130 | 17170260 | 14477702 | 8432543 | 98.2 | 8045817 | 93.7 | 15950550 | 92.9 | 11309811 | 65.9 |
| ME02 | 9662651 | 19325302 | 16008185 | 9530638 | 98.6 | 9146174 | 94.7 | 18198068 | 94.2 | 13637279 | 70.6 |
| WL03 | 10239957 | 20479914 | 16828133 | 10087127 | 98.5 | 9656624 | 94.3 | 19155919 | 93.5 | 13897961 | 67.9 |
| SG00 | 10740395 | 21480790 | 17600834 | 10565061 | 98.4 | 10107894 | 94.1 | 20028136 | 93.2 | 14259544 | 66.4 |
| RC02 | 10323077 | 20646154 | 16888115 | 10172295 | 98.5 | 9715935 | 94.1 | 19272857 | 93.3 | 13649857 | 66.1 |
| KD01 | 10619530 | 21239060 | 17455172 | 10434209 | 98.3 | 9983565 | 94.0 | 19707138 | 92.8 | 13655700 | 64.3 |
| SY03 | 9888370 | 19776740 | 16206132 | 9727195 | 98.4 | 9307415 | 94.1 | 18529567 | 93.7 | 13570627 | 68.6 |
| PA00 | 10018148 | 20036296 | 16455281 | 9881693 | 98.6 | 9466507 | 94.5 | 18855601 | 94.1 | 14091793 | 70.3 |
| MC01 | 9353114 | 18706228 | 15527651 | 9225516 | 98.6 | 8853193 | 94.7 | 17566740 | 93.9 | 12833511 | 68.6 |
| SH00 | 8830010 | 17660020 | 14948002 | 8690924 | 98.4 | 8292527 | 93.9 | 16422149 | 93.0 | 11711400 | 66.3 |
| BR00 | 10599650 | 21199300 | 17811687 | 10436429 | 98.5 | 9979330 | 94.1 | 19805752 | 93.4 | 14520253 | 68.5 |
| HL03 | 10757515 | 21515030 | 17919540 | 10598548 | 98.5 | 10128020 | 94.1 | 20196140 | 93.9 | 15140467 | 70.4 |
| DL19 | 10209180 | 20418360 | 17072768 | 10055948 | 98.5 | 9603570 | 94.1 | 19190374 | 94.0 | 14344168 | 70.3 |
| HP07 | 11008658 | 22017316 | 18346136 | 10850158 | 98.6 | 10369870 | 94.2 | 20701116 | 94.0 | 15555208 | 70.6 |
| CI00 | 11267745 | 22535490 | 18919923 | 11105306 | 98.6 | 10613825 | 94.2 | 21178397 | 94.0 | 15935597 | 70.7 |
| DL13 | 11228219 | 22456438 | 18528979 | 11067838 | 98.6 | 10567846 | 94.1 | 21145067 | 94.2 | 15900599 | 70.8 |
| MD01 | 9558354 | 19116708 | 16181341 | 9406699 | 98.4 | 8969971 | 93.8 | 17840363 | 93.3 | 13073506 | 68.4 |
| CC11 | 11517038 | 23034076 | 19083911 | 11374094 | 98.8 | 10885813 | 94.5 | 21735526 | 94.4 | 16417241 | 71.3 |
| SG02 | 10377812 | 20755624 | 17348615 | 10208967 | 98.4 | 9711916 | 93.6 | 19308692 | 93.0 | 13751921 | 66.3 |
| ME00 | 10143667 | 20287334 | 17028840 | 9998746 | 98.6 | 9545666 | 94.1 | 19042691 | 93.9 | 14153117 | 69.8 |
| GW03 | 8918330 | 17836660 | 15217757 | 8791373 | 98.6 | 8392263 | 94.1 | 16757448 | 93.9 | 12491350 | 70.0 |
| PA01 | 9658212 | 19316424 | 16369562 | 9526329 | 98.6 | 9099215 | 94.2 | 18181053 | 94.1 | 13594931 | 70.4 |
| NA06 | 10312065 | 20624130 | 17305915 | 10173215 | 98.7 | 9719347 | 94.3 | 19425344 | 94.2 | 14360565 | 69.6 |
| HB06 | 9898102 | 19796204 | 16746477 | 9747490 | 98.5 | 9316055 | 94.1 | 18522136 | 93.6 | 13261326 | 67.0 |
| BF02 | 9707917 | 19415834 | 16535894 | 9573686 | 98.6 | 9161289 | 94.4 | 18237821 | 93.9 | 13493900 | 69.5 |
| LI03 | 10097352 | 20194704 | 17044019 | 9964409 | 98.7 | 9522682 | 94.3 | 19005445 | 94.1 | 14080842 | 69.7 |
| DE10 | 8622338 | 17244676 | 14897145 | 8503443 | 98.6 | 8145354 | 94.5 | 16195055 | 93.9 | 11979458 | 69.5 |
| PA08 | 11119431 | 22238862 | 18483101 | 10975675 | 98.7 | 10522723 | 94.6 | 20996776 | 94.4 | 15643661 | 70.3 |
| SU12 | 9960798 | 19921596 | 16870946 | 9841254 | 98.8 | 9435019 | 94.7 | 18814811 | 94.4 | 14011425 | 70.3 |
| WH07 | 11557464 | 23114928 | 19052597 | 11401905 | 98.7 | 10913932 | 94.4 | 21811588 | 94.4 | 16172156 | 70.0 |
| SU17 | 10282431 | 20564862 | 17264575 | 10150954 | 98.7 | 9708418 | 94.4 | 19420423 | 94.4 | 14477020 | 70.4 |
| RR01 | 10418489 | 20836978 | 17401572 | 10276289 | 98.6 | 9804544 | 94.1 | 19558530 | 93.9 | 13997366 | 67.2 |
| CW06 | 8640639 | 17281278 | 14893221 | 8533953 | 98.8 | 8173963 | 94.6 | 16321577 | 94.4 | 12175398 | 70.5 |
| HL01 | 10078471 | 20156942 | 17024701 | 9929782 | 98.5 | 9460667 | 93.9 | 18855017 | 93.5 | 13630762 | 67.6 |
| AC02 | 9138646 | 18277292 | 15383418 | 8979725 | 98.3 | 8549708 | 93.6 | 16886409 | 92.4 | 11618665 | 63.6 |
| KD13 | 9217525 | 18435050 | 15430700 | 9062536 | 98.3 | 8657895 | 93.9 | 17025100 | 92.4 | 11577542 | 62.8 |
| BF04 | 9190330 | 18380660 | 15233896 | 9073948 | 98.7 | 8710876 | 94.8 | 17346321 | 94.4 | 13018548 | 70.8 |
| KL03 | 9875945 | 19751890 | 16216100 | 9749246 | 98.7 | 9354125 | 94.7 | 18629510 | 94.3 | 13919070 | 70.5 |
| NB01 | 7780722 | 15561444 | 13268565 | 7669803 | 98.6 | 7341275 | 94.4 | 14600345 | 93.8 | 10860490 | 69.8 |
| CC12 | 9517671 | 19035342 | 15806695 | 9389409 | 98.7 | 9008011 | 94.6 | 17872062 | 93.9 | 13261568 | 69.7 |
| AC05 | 9521769 | 19043538 | 15889298 | 9373608 | 98.4 | 8953422 | 94.0 | 17643059 | 92.6 | 12132184 | 63.7 |
| DL05 | 9811519 | 19623038 | 16091805 | 9687029 | 98.7 | 9288568 | 94.7 | 18503531 | 94.3 | 13857700 | 70.6 |
| SV08 | 8768763 | 17537526 | 15229821 | 8516154 | 97.1 | 8127795 | 92.7 | 15495843 | 88.4 | 8580669 | 48.9 |
| SA00 | 9940187 | 19880374 | 16408795 | 9758550 | 98.2 | 9339561 | 94.0 | 18569142 | 93.4 | 13787511 | 69.4 |
| SF00 | 8331246 | 16662492 | 14078006 | 8209985 | 98.5 | 7858656 | 94.3 | 15527998 | 93.2 | 11004110 | 66.0 |
| GW19 | 8931795 | 17863590 | 14866174 | 8799776 | 98.5 | 8426374 | 94.3 | 16775528 | 93.9 | 12326599 | 69.0 |
| ***min*** | ***7780722*** | ***15561444*** | ***13268565*** | ***7669803*** | ***97.1*** | ***7341275*** | ***92.7*** | ***14600345*** | ***88.4*** | ***8580669*** | ***48.9*** |
| ***max*** | ***11557464*** | ***23114928*** | ***19083911*** | ***11401905*** | ***98.8*** | ***10913932*** | ***94.8*** | ***21811588*** | ***94.4*** | ***16417241*** | ***71.3*** |
