# Supplementary figures and images for: Targeted enrichment of the black cottonwood (Populus trichocarpa) gene space using sequence capture
Source: BMC Genomics. 2012 Dec 14;13:703. doi: 10.1186/1471-2164-13-703 (PMC3542275; doi:10.1186/1471-2164-13-703)

## Coverage of regions adjacent to baits

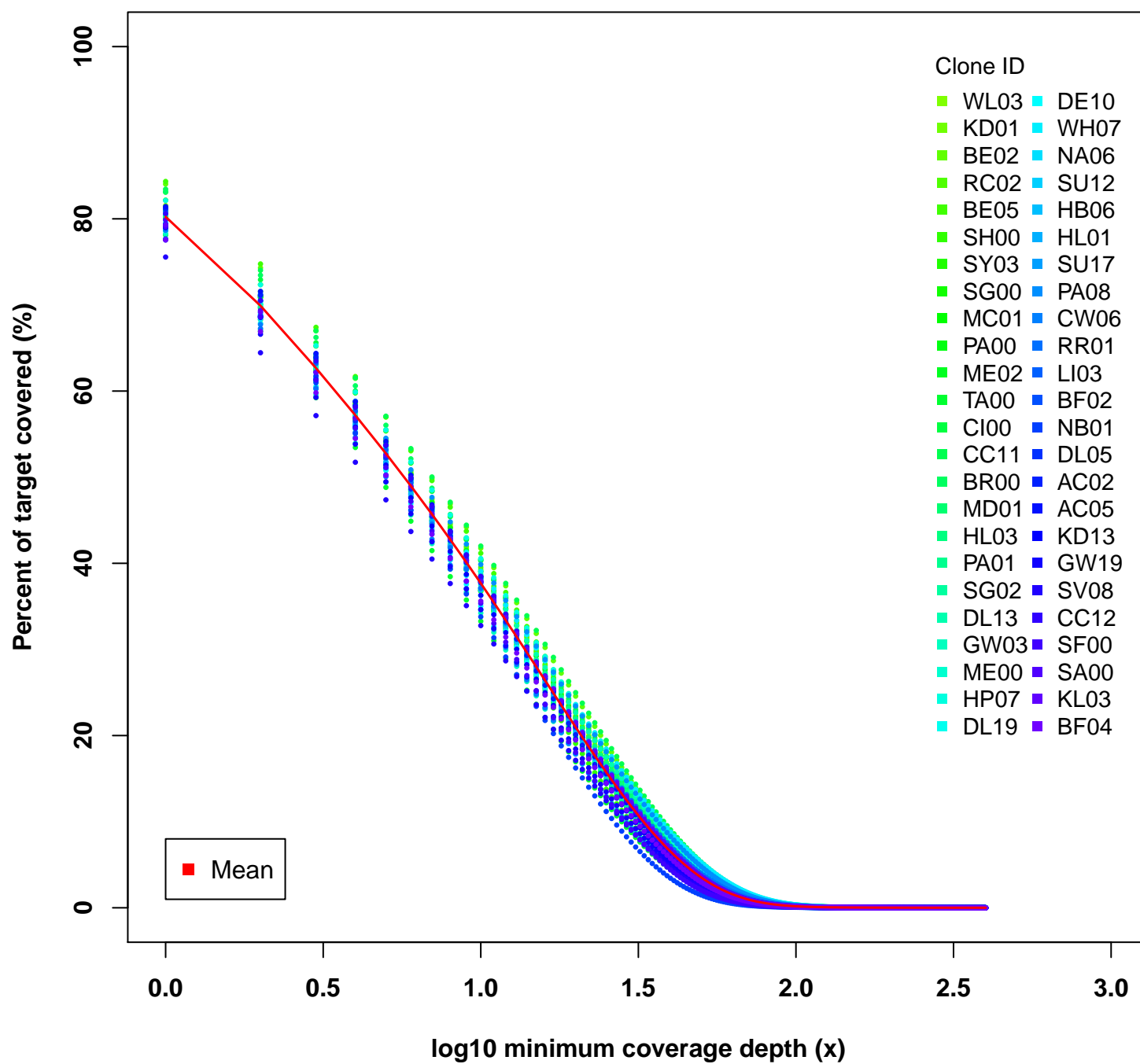

Supplement: Additional file 3 — Figure S1. Cumulative distribution of coverage depth in adjacent regions adjacent to baits. Both mean coverage across all 48 genotypes (red line) and mean coverage for individual genotypes (colored points) are provided. [file 1471-2164-13-703-S3.pdf]

## Coverage of off-bait regions

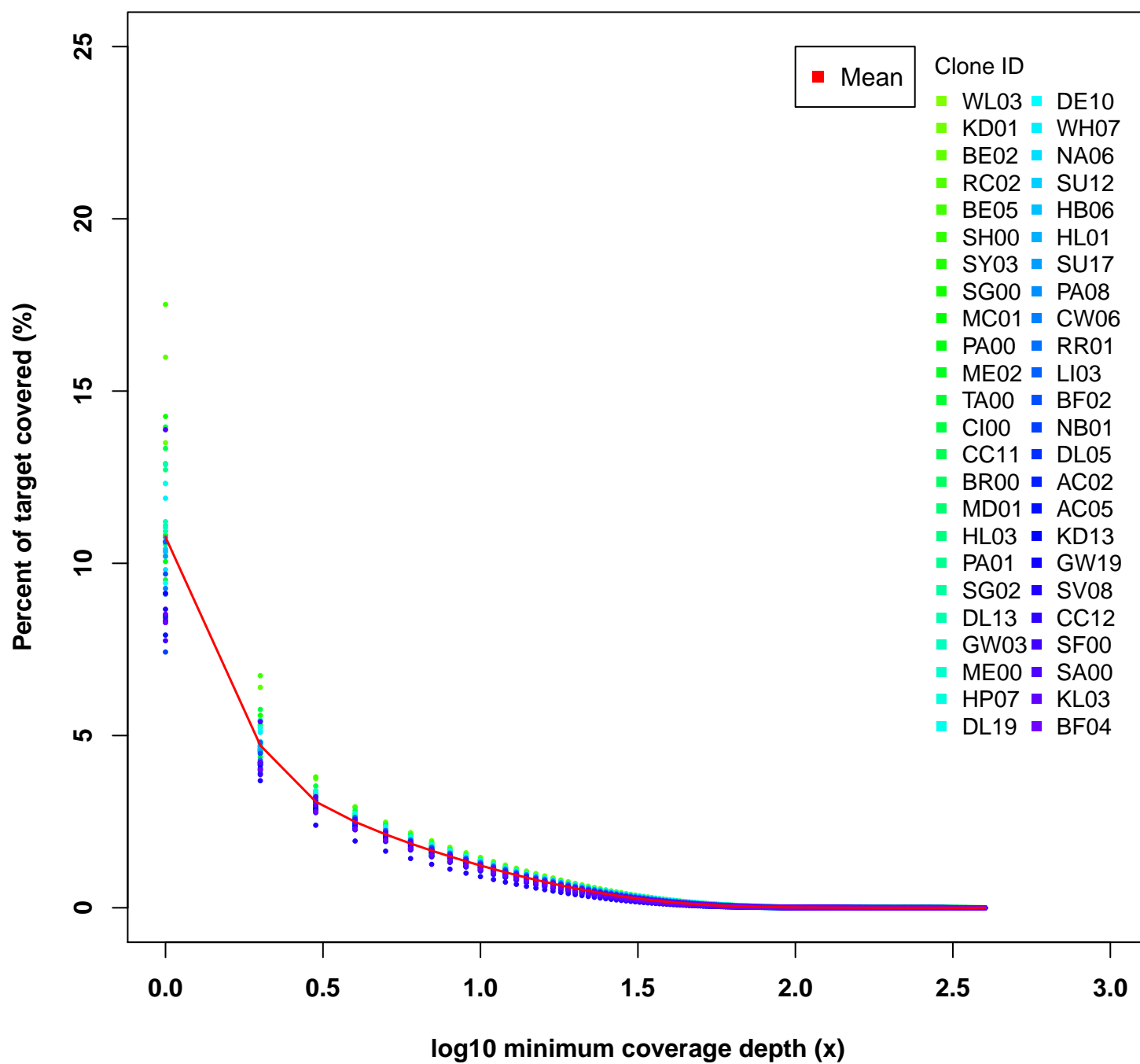

Supplement: Additional file 4 — Figure S2. Cumulative distribution of coverage depth in off-target regions. Both mean coverage across all 48 genotypes (red line) and mean coverage for individual genotypes (colored points) are provided. [file 1471-2164-13-703-S4.pdf]

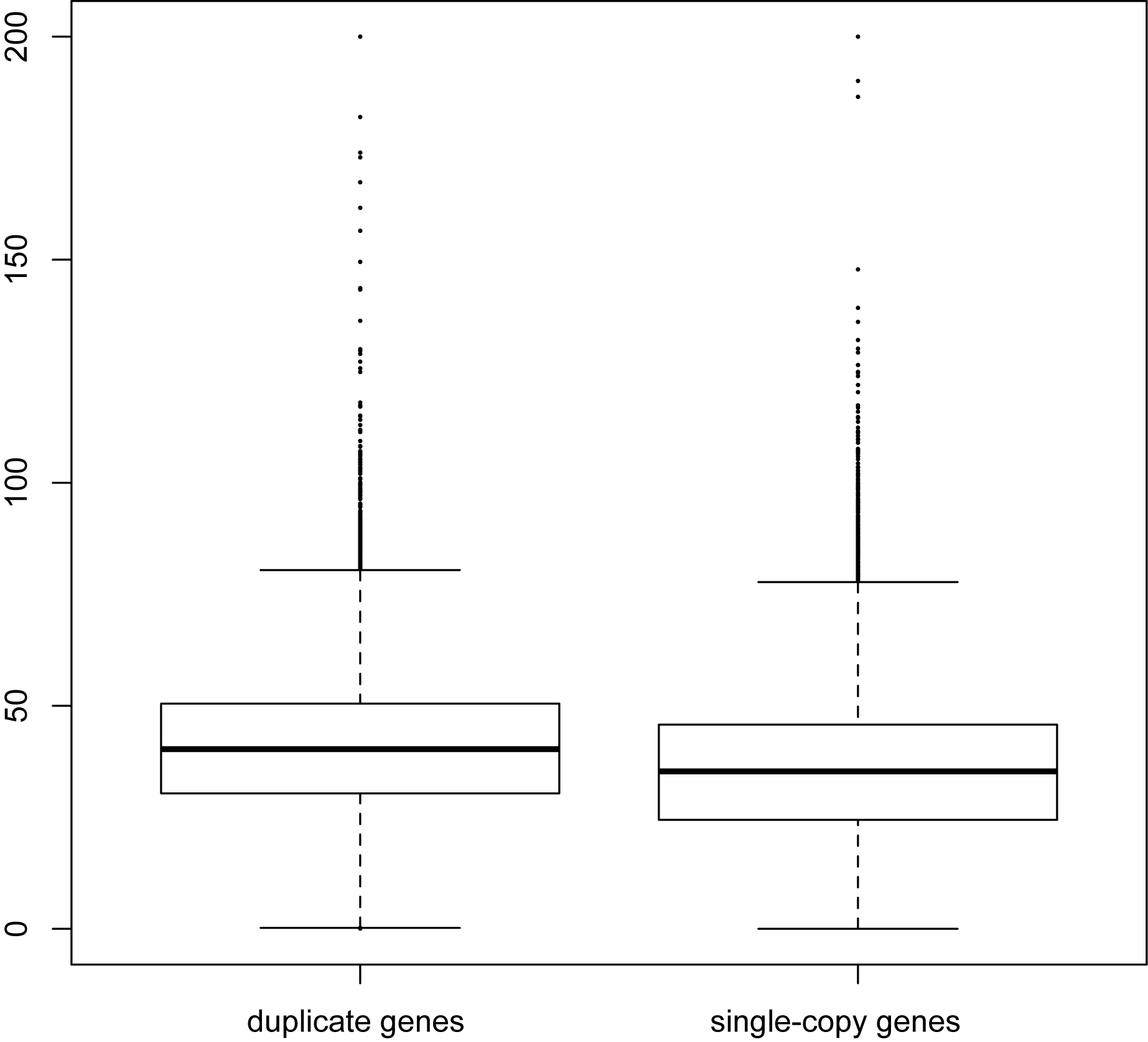

Supplement: Additional file 5 — Figure S3. Boxplots of coverage depth for single-copy genes and those with retained salicoid duplicates. [file 1471-2164-13-703-S5.tiff]

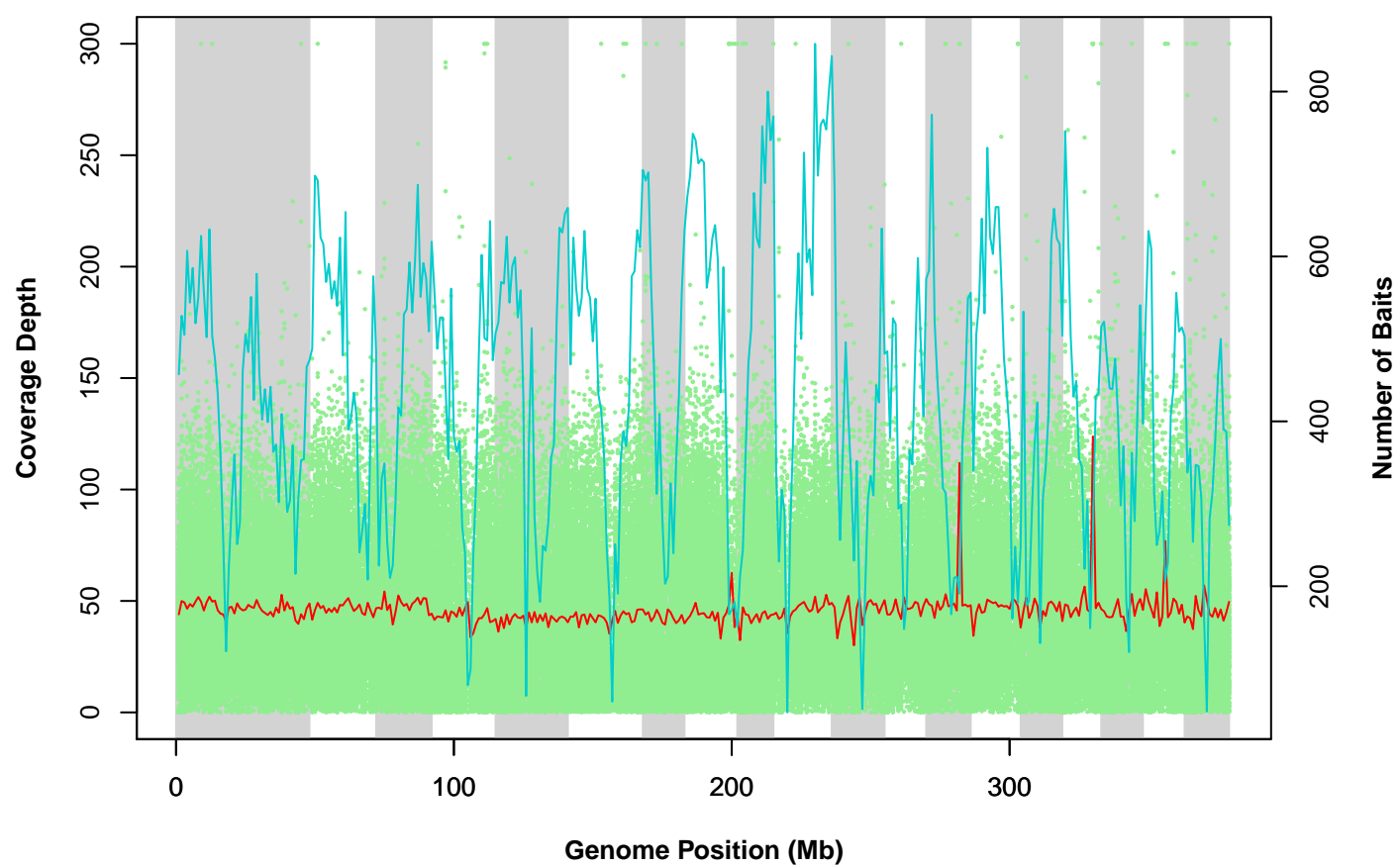

Supplement: Additional file 6 — Figure S4. Distribution of baits across the 19 poplar linkage groups. Points indicate bait locations, light blue line indicates number of baits in 1Mb sliding windows, and red line indicates mean depth of sequencing coverage in 1Mb sliding windows. [file 1471-2164-13-703-S6.pdf]
